# Plasma GFAP in presymptomatic and symptomatic familial Alzheimer’s disease: a longitudinal cohort study

**DOI:** 10.1136/jnnp-2022-329663

**Published:** 2022-08-10

**Authors:** Antoinette O'Connor, Emily Abel, Andrea Lessa Benedet, Teresa Poole, Nicholas Ashton, Philip Simon John Weston, Amanda J Heslegrave, Natalie Ryan, Suzie Barker, James M Polke, Kaj Blennow, Henrik Zetterberg, Nick C Fox

**Affiliations:** 1 Dementia Research Centre, UCL Queen Square Institute of Neurology, London, UK; 2 UK Dementia Research Institute at UCL, London, UK; 3 Department of Psychiatry and Neurochemistry, Institute of Neuroscience and Physiology, Sahlgrenska Academy at University of Gothenburg, Mölndal, Sweden; 4 Department of Medical Statistics, London School of Hygiene & Tropical Medicine, London, UK; 5 Centre for Age-Related Medicine, Stavanger University Hospital, Stavanger, Norway; 6 NIHR Biomedical Research Centre for Mental Health & Biomedical Research Unit for Dementia, South London & Maudsley NHS Foundation Trust, London, UK; 7 King's College London Institute of Psychiatry, Psychology and Neuroscience, Maurice Wohl Clinical Neuroscience Institute, London, UK; 8 Neurogenetics Laboratory, National Hospital for Neurology and Neurosurgery, University College London NHS Foundation Trust, London, UK; 9 Clinical Neurochemistry Laboratory, Sahlgrenska University Hospital, Mölndal, Sweden

**Keywords:** alzheimer's disease, neurochemistry

## Introduction

Glial fibrillar acidic protein (GFAP), a marker of astroglia activation, has been proposed as a biomarker of Alzheimer’s disease (AD).^1^ GFAP expression correlates with Aβ plaque density and cerebrospinal fluid (CSF) concentration is elevated in symptomatic disease.^1 2^ Ultrasensitive assays that reliably measure plasma GFAP show increases in AD that are relatively greater than in CSF.^1^ Autosomal dominantly inherited familial AD (FAD) is a valuable model for characterising presymptomatic AD as mutations are highly penetrant and it has a young, reasonably predictable, age of onset.^3^ We examined whether plasma GFAP concentration is altered in mutation carriers compared with non-carriers, and the timing of presymptomatic change.

## Methods

We studied 69 participants within University College London’s longitudinal study of FAD between 2010 and 2019; described previously.^3^ Eligibility was either (1) a clinical diagnosis of FAD or (2) an FAD-affected parent, which means a 50% risk of inheriting a mutation and thereby of developing symptoms at a similar age to their affected parent.

FAD mutation status was determined using Sanger sequencing; participants and study clinicians were blinded to results. At each study visit, EDTA blood sampling and a participant and informant interview were conducted. Plasma samples were shipped frozen to Sahlgrenska University Hospital for blinded analysis using the GFAP single molecule array discovery kit (#102336) on an HD-X platform (Quanterix). Estimated years to/from symptom onset (EYO) was calculated by subtracting the age at which the participant’s affected parent first developed progressive cognitive symptoms from the participant’s age at blood sampling.

Baseline statistics and box plots of GFAP concentrations were produced for each participant group (symptomatic mutation carriers; presymptomatic carriers; non-carriers). Other analyses used data from all visits. GFAP was log-transformed, with estimated coefficients back-transformed and expressed as multiplicative effects and geometric means. In non-carriers we assessed the association between GFAP and sex. Age-adjusted and sex-adjusted (1) differences in GFAP between patient groups and (2) relationship between GFAP and EYO were both modelled using mixed effects models. We estimated the age-adjusted and sex-adjusted difference in geometric mean GFAP between carriers and non-carriers for integer values of EYO between –30 and 20. The point when this estimate was statistically significantly different from zero (p≤0.05) was interpreted cautiously as an indication of when the estimated trajectory of GFAP for carriers diverged from non-carriers. A sensitivity analysis refitted all models omitting participants (one symptomatic mutation carrier, one non-carrier) with high outlier GFAP values.

Further details on study procedures and analyses are provided in online supplemental material.

10.1136/jnnp-2022-329663.supp1 Supplementary data

## Results

**Figure 1:**
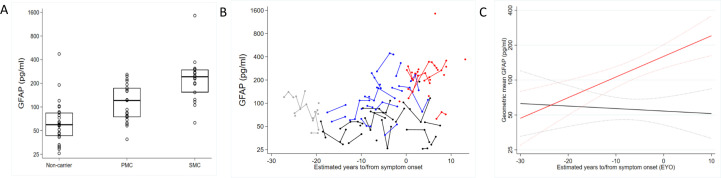
(A) Box plot for observed baseline plasma GFAP concentration. The measured plasma GFAP concentrations at baseline (first visit) are shown. Mutation carriers have been divided into those who are symptomatic (SMC) and those who are presymptomatic (PMC). The estimated geometric mean GFAP concentration in symptomatic carriers was 238% higher (95% CI 141% to 374%, p<0.001) than in non-carriers, and in presymptomatic carriers was 95% higher (95% CI 46% to 159%, p<0.001) than in non-carriers, after adjusting for age and sex. Age-and sex-adjusted geometric mean GFAP concentration was also significantly elevated in symptomatic compared with presymptomatic carriers (73% higher, 95% CI 26% to 138%, p=0.001). Boxes show the median and first and third quartiles. Dots represent individual observations. (B) Observed plasma GFAP concentration against estimated years to/from symptom onset. Symptomatic mutation carriers are shown in red, presymptomatic mutation carriers are shown in blue, non-carriers are shown in black. To preserve blinding to genetic status, all observed values for timepoints more than 19.3 years before expected symptom onset are shown in grey and some timepoints have been removed for at risk individuals. (C) Trajectory of plasma GFAP against estimated years to/from onset. Modelled geometric mean plasma against estimated years to/from symptom onset. Mutation carriers represented in red; non-carriers in black. Estimates are shown for a hypothetical male aged 41 years (the mean age at baseline). Dotted lines indicate 95% CIs. The y-axis scale is logarithmic in all panes. GFAP, glial fibrillar acidic protein.

Online supplemental table 1 shows baseline characteristics. Fifty participants were asymptomatic (23 mutation carriers 27 non-carrier controls). Baseline and longitudinal observed GFAP data are shown in figure 1A, B. Within non-carriers, estimated geometric mean plasma GFAP was higher in females compared with males (54% higher, 95% CI 2% to 133%, p=0.039), with no meaningful difference after omitting the outlier. After adjusting for age at visit and sex, geometric mean GFAP concentrations were estimated to be higher in both symptomatic and presymptomatic carriers compared with non-carriers (p<0.001 for both comparisons). These results remained significant after removing the two outliers (online supplemental figure 1). The age- and sex-adjusted geometric mean GFAP concentration in carriers was first significantly higher (p=0.04) than in non-carriers at EYO of 16 years (figure 1C).

## Discussion

This study found plasma GFAP was increased in both presymptomatic and symptomatic mutation carriers compared with non-carriers, and in symptomatic compared with presymptomatic carriers. Plasma GFAP levels diverged between carriers and non-carriers around 16 years before estimated symptom onset. This is consistent with recent findings of higher plasma GFAP in amyloid-positive versus amyloid-negative cognitively normal older adults and with GFAP increases being associated with subsequent decline in global cognition, amyloid accumulation and conversion to dementia.^1 2 4^ Overall these results support plasma GFAP being a biomarker of early AD pathology.

The timing of GFAP change is consistent with a response to amyloid, supporting previous findings that plasma GFAP is associated with amyloid burden and p-tau181 levels.^1 2^ Additionally, plasma levels partially mediate the association between amyloid burden and tau positron emission tomography (PET) signal, and are not increased in amyloid negative, tau positive individuals or in dementia with Lewy bodies and frontotemporal dementia.^1 2^ This suggests that plasma GFAP is a marker of amyloid-related astrogliosis. However, plasma GFAP may also be an indicator of blood–brain barrier and/or glymphatic dysfunction—astrocytes form part of the neurovascular unit and may directly release GFAP into blood, perhaps explaining the greater magnitude of increases in plasma versus CSF GFAP, and the lack of association between plasma GFAP and other CSF inflammatory markers.^1 2 5^

Symptomatic carriers, on average, had geometric mean plasma GFAP concentrations greater than three times that of non-carriers, with presymptomatic carriers having a mean concentration twice that of non-carriers, approaching midway between symptomatic and non-carrier groups. These are remarkable differences given this is a blood-based assay. Nonetheless, the overlap between groups, the outliers and the within-individual variability suggests that plasma GFAP may be most useful in combination with other AD blood biomarkers. This is consistent with recent studies in sporadic AD showing higher diagnostic yield for detecting underlying amyloid/AD pathology when GFAP was used in combination with other plasma markers.^1^

Plasma GFAP demonstrated considerable intraindividual and interindividual variability; fluctuations in plasma concentrations of NfL and p-tau181 have previously been shown in the same cohort.^3^ It is unlikely that AD-related processes are solely responsible for these fluctuations as variability occurred in carrier and non-carrier groups. Variability in plasma levels may be partly attributable to responses to nonspecific CNS injury, with increases previously being reported in traumatic brain injury, stroke, epilepsy and COVID-19-related delirium.^5^

Our study has limitations. The sample size, due to the rarity of FAD, was relatively small. Additionally, we used parental age at symptom onset to estimate timing of future symptomatic decline. This provides a reasonable estimate of future age at onset, but it is not without error due to variability in age at onset. Further, prospective studies are needed to assess diagnostic accuracy and investigate sources of variability.

## Conclusion

Plasma GFAP concentration in FAD increases presymptomatically, with changes being detected over a decade prior to estimated symptom onset, supporting its further investigation as an accessible biomarker of AD-related astroglial activation.
