# Supplementary material for: Plasma GFAP in presymptomatic and symptomatic familial Alzheimer’s disease: a longitudinal cohort study
Source: J Neurol Neurosurg Psychiatry. 2022 Aug 10;94(1):90–2. doi: 10.1136/jnnp-2022-329663 (PMC9763156; doi:10.1136/jnnp-2022-329663)
Supplement: Supplementary data [file jnnp-2022-329663supp001.pdf]

Methods

Study Procedures

All individuals identified an informant who was interviewed separately for a collateral history. At each study visit, a semi-structured health questionnaire (including exclusion of recent head injury), neurological examination, and the clinical dementia rating (CDR) scale were performed (1). Individuals were defined as symptomatic if cognitive decline was reported by the participant and/or their informant and the global CDR was >0.

GFAP quantification in plasma

Samples were collected, processed, aliquoted, and frozen at −80 °C according to standardised procedures. Non-fasting plasma samples were collected in 10 ml ethylenediaminetetraacetic acid coated tubes. Prior to analysis plasma samples were vortexed (2000rpm) and centrifugated (4000*g*, 10minutes) at room temperature. An 8-fold dilution was used. The intra- and inter-assay variation of internal quality control samples were <10%.

Statistical analysis

Age- and sex-adjusted differences in GFAP between patient groups were estimated using a mixed effects model that included random intercepts for individual and family membership, with residual variances differing for the groups being compared. Two presymptomatic individuals became symptomatic during follow-up, with their group membership changed appropriately in analyses.

The mixed effects model that examine the age- and sex-adjusted relationship between GFAP and EYO included: a random intercept for family; random intercept and slope for EYO at individual level allowing these to differ by genetic mutation status; residual variances differing by mutation status. We pre-specified inclusion of mutation status and EYO, plus their interaction, as fixed effects. We investigated quadratic and cubic terms for EYO, plus their interactions with mutation status, but there was no evidence to include these terms. The estimated geometric mean longitudinal GFAP concentration trajectories for mutation

carriers and non-carriers (and 95% confidence intervals) were plotted against EYO, standardised to a male aged 41 years (mean baseline age of all participants).

Analyses used Stata v17.

Supplementary Table 1

	**Non-carrier**	**PMC**	**SMC**
**N**	27	23	19
**Sex, n (%)**<br>**Women**<br>**Men**	 16 (59%)<br>11 (41%)	 11 (48%)<br>12 (52%)	 7 (37%)<br>12 (63%)
**Age, years (mean (SD))**	38.1 (10.7)	35.7 (5.8)	51.3 (9.5)
**EYO, years (mean (SD))**	N/A	-10.4 (7.6)	4.2 (3.8)
**MMSE (Median [IQR])**	30 [30, 30]	30 [29, 30]	23 [16, 25][#]
**CDR Global (Median [IQR])**	0 [0, 0]	0 [0, 0]	0.5 [0.5, 0.75] (n = 16)
**CDR SOB (Median [IQR])**	0 [0, 0]	0 [0, 0]	3.5 [1.75, 4.25] (n = 16)
**GFAP follow-up duration, years (mean, (SD), for participants with more than one visit)**	3.0 (2.0) (n=17)	3.1 (1.8) (n=17)	3.5 (2.0) (n=6)
**Samples per participant (mean)***	2.1	2.4	1.8

**GFAP, pg/ml (mean (SD))**	80.2 (85.7)	127.9 (66.2)	287.4 (293.3)

* Forty of the overall 69 participants had at least two visits.
#Of the symptomatic group, two had severe AD (MMSE <10), five had moderate AD (MMSE 10-20), and 12 had mild AD (MMSE > 20).

Supplementary Figure 1

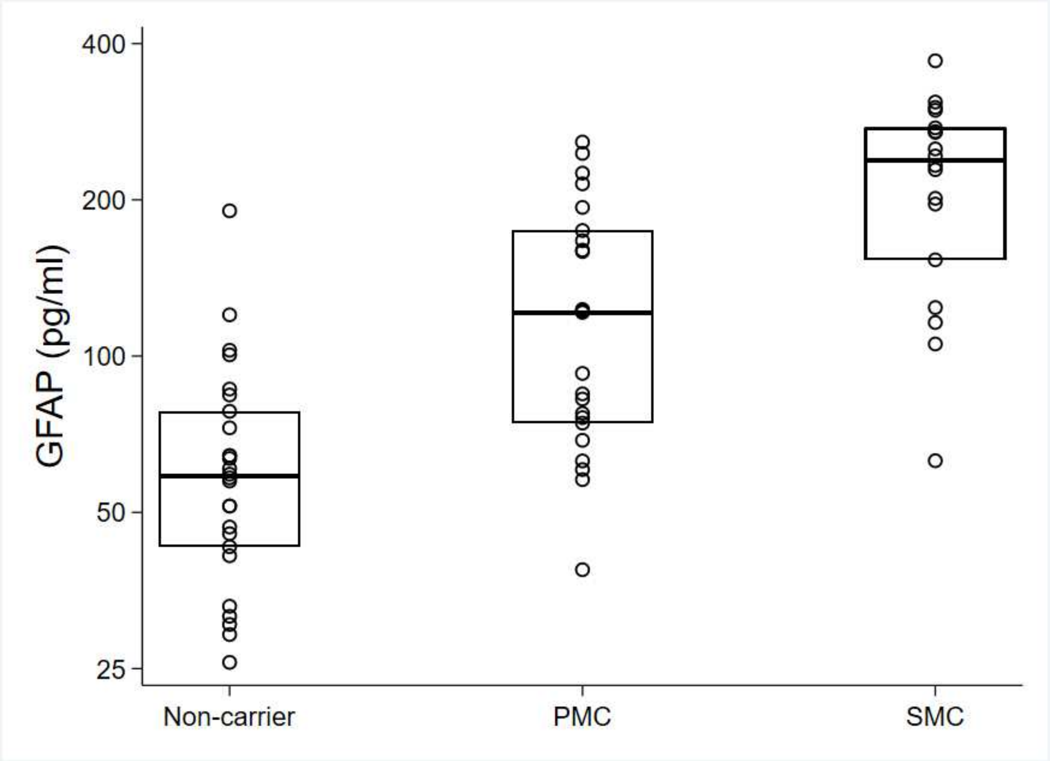

Box plot for observed baseline plasma GFAP concentrations with outliers removed. *The measured plasma GFAP concentrations at baseline (first visit) are shown. Mutation carriers have been divided into those who are symptomatic (SMC) and those who are presymptomatic (PMC). Two high outliers (one SMC, one non-carrier; each with one observation) have been removed. After adjusting for age and sex, geometric mean GFAP is estimated to be 218% higher (95%CI: 137%, 328%) in SMC compared to non-carriers (p<0.001) and is estimated to be 104% higher (95%CI: 60%, 160%) in PMC compared to non-carriers (p<0.001). Additionally after adjusting for age and sex, geometric mean GFAP is an estimated 56% higher (95%CI: 17%, 108%) in SMC compared to PMC (p=0.002).Boxes show the median and first and third quartiles. Dots represent individual observations. The y-axis scale is logarithmic.*

References

1. Morris JC. The Clinical Dementia Rating (CDR): Current version and scoring rules. Neurology [Internet]. 1993 Nov 1 [cited 2018 Feb 21];43(11):2412–2412. Available from: http://www.ncbi.nlm.nih.gov/pubmed/8232972
